# Polar Organic Gate Dielectrics for Graphene Field-Effect Transistor-Based Sensor Technology

**DOI:** 10.3390/s18092774

**Published:** 2018-08-23

**Authors:** Kevin A. Kam, Brianne I. C. Tengan, Cody K. Hayashi, Richard C. Ordonez, David G. Garmire

**Affiliations:** 1 Department of Electrical Engineering, University of Hawai’i at Manoa, Honolulu, HI 96822, USA; kamkevin@hawaii.edu (K.A.K.); briannet@hawaii.edu (B.I.C.T.); 2 Space and Naval Warfare Systems Command of the Pacific, Pearl City, HI 96782, USA; cody.hayashi@navy.mil (C.K.H.); richard.c.ordonez@navy.mil (R.C.O.)

**Keywords:** graphene field-effect transistors, polar organic dielectrics, flexible graphene-based sensor technology

## Abstract

We have pioneered the use of liquid polar organic molecules as alternatives to rigid gate-dielectrics for the fabrication of graphene field-effect transistors. The unique high net dipole moment of various polar organic molecules allows for easy manipulation of graphene’s conductivity due to the formation of an electrical double layer with a high-capacitance at the liquid and graphene interface. Here, we compare the performances of dimethyl sulfoxide (DMSO), acetonitrile, propionamide, and valeramide as polar organic liquid dielectrics in graphene field-effect transistors (GFETs). We demonstrate improved performance for a GFET with a liquid dielectric comprised of DMSO with high electron and hole mobilities of 154.0 cm^2^/Vs and 154.6 cm^2^/Vs, respectively, and a Dirac voltage <5 V.

## 1. Introduction

Progress into the realization of flexible electronic devices has been hampered by the lack of robust dielectric materials that maintain a desirable electrical performance under high strain. Traditionally, gate dielectrics for field-effect transistors (FETs) are fabricated with thin layers of aluminum, hafnium, or silicon dioxide [1]. While these oxides exhibit a variety of advantages as noted by their predominant use in today’s commercial industry, they possess several limitations for applications in flexible sensors. Due to the solid state of these thin-film oxides, there is a nominal mechanical failure threshold under high strain [1]. Furthermore, the oxide’s dielectric constant is directly affected by strain, which can produce significant variability in performance [2].

Recent research in flexible electronics has explored the utilization of two-dimensional graphene in thin-film devices due to its high carrier mobility, high tensile strength, and broadband sensing capability [3,4]. Graphene is defined as a monolayer of carbon atoms arranged in a hexagonal lattice with a bond length of 1.42 Å and a lattice constant of 2.46 Å [5]. The unique atomic structure of graphene gives rise to its exceptional electrical and mechanical properties that enable a plethora of potential applications for next-generation wearable devices, transducers, and sensors. However, due to the lack of flexible materials for gate dielectrics, graphene field-effect transistors (GFETs) have been constrained by limitations similar to that of traditional flexible FETs [5]. As flexible devices become a trademark in the commercial sector, there is a need for flexible gate dielectric materials in order realize robust wearable devices with flexible GFETs.

In this work, we demonstrate several advantages of the use of high-k polar organic liquid dielectrics when implemented in flexible GFETs. Given the liquid form of polar organic dielectrics, these materials become self-healing under strain and will conform neatly to unconventional surface geometries [6]. Unlike traditional gate oxide materials, the electrical performance of polar organic dielectrics has been shown to be independent of the physical thickness of the dielectric [7]. Therefore, introducing a dielectric material that is robust under strain in terms of mechanical failure and performance variation will vastly maintain and potentially improve the GFET’s electrical characteristics under flexing. Additionally, these liquid dielectrics possess electrical advantages over their solid counterparts, including higher dielectric breakdown voltages and significantly lower operating voltages [1] making these devices easier to integrate with current semiconductor technology. Finally, the introduction of these liquid dielectrics enables researchers to overcome barriers-to-entry in the fabrication of flexible GFET devices. Instead of requiring expensive machinery and highly-trained technicians for atomic layer deposition (ALD) and chemical vapor deposition techniques utilized in oxide growth, researchers could utilize inexpensive drop-casting and/or spin-coating methods [8].

**Figure 1:**
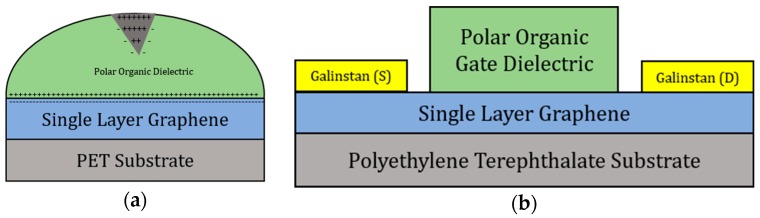
(**a**) Device layout showing the electric double layer being induced at the graphene-organic dielectric interface; (**b**) transistor architecture block diagram of the polar organic gated graphene field-effect transistors (GFETs).

The ability of these organic compounds to exhibit dielectric characteristics is due to their intrinsic molecular polarity. Polarity in organic compounds arises from the difference in electronegativity between two covalently bonded atoms. When exposed to an electric field, the partial charges on the molecules’ resonance structures orient themselves, creating an electric double layer at the graphene-dielectric interface [9,10]. Shown in Figure 1a, the gate dielectric is probed with a gate voltage that causes the molecules to align, creating a gating effect in the transistor.

**Figure 2:**
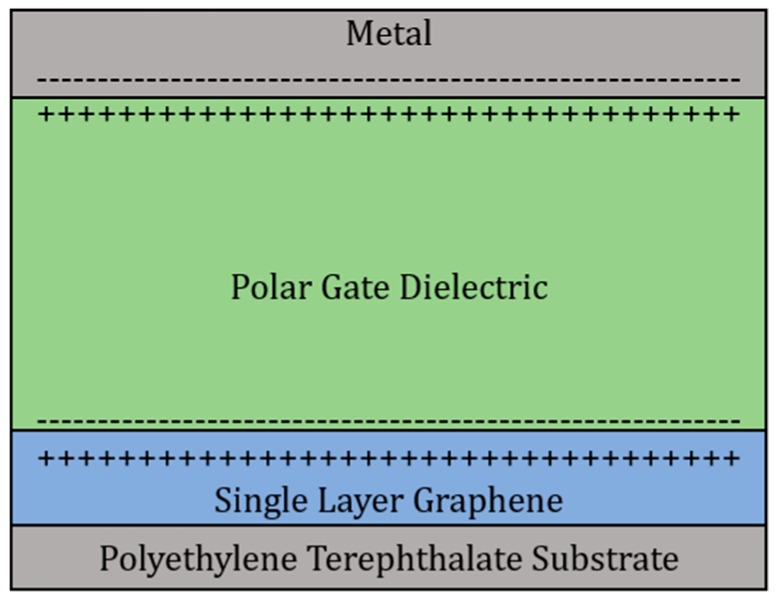
Illustration of the electric double layer at the polar dielectric-graphene interface.

Figure 2 illustrates the formation of an electric double layer in more detail. When a voltage is applied to the gate electrode, ions from the dielectric are attracted to the charge on the gate. The electrostatic accumulation of charges on either side of the dielectric acts as an electric double layer capacitor and induces an electric field at the graphene-dielectric interface. Studies on the graphene electric double layer-graphene interface have shown the quantum capacitance is due to the density of states of graphene [11]. This quantum capacitance value increases as more layers of graphene are implemented [11].

To study the phenomenon that creates this field-effect, four polar organic molecules were integrated with graphene transistors. The organic molecules DMSO, acetonitrile, propionamide, and valeramide were chosen due to their high dipole moments and permittivities, which make them ideal gate dielectrics for flexible GFET devices by allowing for the creation of a large field-effect with the application of a voltage bias.

## 2. Materials and Methods

### 2.1. Device Fabrication

The GFETs were fabricated with the methods shown in [10]. Single-layer graphene on a polyethylene terephthalate (PET) substrate was purchased from Graphene Supermarket. A strip of graphene on PET was cut and adhered on a microscope slide using double-sided polyimide tape, PET side down. A liquid polar organic molecule was drop-casted within the graphene channel using a 1 mL syringe with a 27 G needle attached. To overcome the high contact resistance of graphene [4], Galinstan, a commercially available eutectic alloy comprising of 68% gallium, 22% indium, and 10% tin, was used as the source, drain, and gate electrodes. Galinstan exhibits a desirable conductivity (2.30 × 10^6^ S/m), is found in its liquid phase over a large temperature range (−19 °C to 1300 °C), and has a moderate wettability to graphene which reduces damage to the graphene material under test [4].

**Figure 3:**
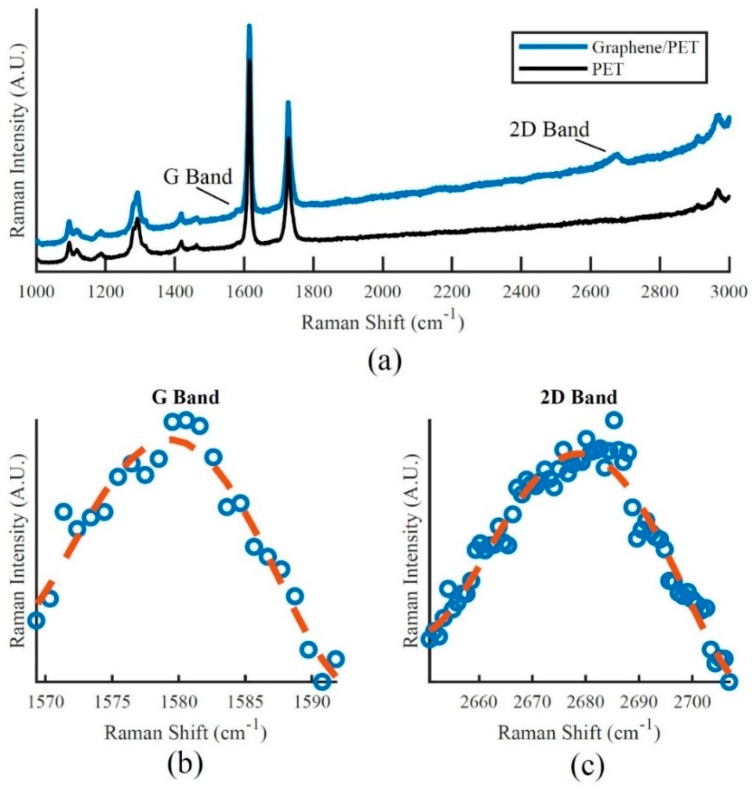
(**a**) Raman spectroscopy profile of monolayer graphene on Polyethylene Terephthalate (PET) and location of the Raman peaks of graphene; (**b**) Raman spectroscopy profile of graphene G-Band peak and (**c**) Raman spectroscopy profile of graphene 2D Band peak.

To verify the quality of the monolayer graphene sample, 514.5 nm Raman spectroscopy measurements were conducted with a Renishaw Invia Micro-Raman System. It is important to note, the G-Band and the 2D band for monolayer graphene on PET are difficult to measure as there are strong polymeric vibrational modes that are sensitive to Raman scattering near the G-Band of graphene [5], see Figure 3a. Therefore to get an accurate measurement of the graphene Raman peaks on PET, one needs to operate the Raman spectroscopy system in a static mode to improve the resolution, see Figure 3b,c.

Once the Raman measurements were taken for the graphene sample on PET, post experimental data processing was also performed to remove the scattering effects of the PET substrate. The I_2D_/I_G_ ratio for the graphene samples was determined to be greater than 2.23 which proves the presence of monolayer graphene on the samples used.

### 2.2. Dielectric Synthesis

DMSO, acetonitrile, propionamide, and valeramide were purchased from Sigma Aldrich. While DMSO and acetonitrile were available as pure aqueous solutions, propionamide and valeramide were only available in a powder form and needed to be mixed with deionized water before deposition on the devices. To synthesize the propionamide solution, 50 mg of propionamide was weighed using a weigh-boat on a Mettler Toledo Microbalance. A quantity of 30 μL of deionized water was then added via a Cole-Parmer 0.1–10 μL micropipette, creating an aqueous amide solution of propionamide with a weight/volume concentration of 1.67 g/mL. The final solution was then drop-casted onto the graphene channel as stated in Section 2.1. The valeramide solution was synthesized in a similar fashion, but with 30.6 mg of valeramide and 83.8 μL of deionized water, which yielded a weight/volume concentration of 0.37 g/mL. Due to the aqueous nature of acetonitrile and DMSO, no deionized water was needed and the solutions were pipetted directly from their stock containers.

### 2.3. Dielectric Characterization

The dielectric constant for the aqueous amide solutions of propionamide and valeramide were experimentally verified with a parallel plate capacitor method consisting of two 10 mm × 20 mm Bertech copper conductive tapes serving as conducting plates. These plates were placed 4 mm apart and loaded with the 800 mm^3^ of the dielectrics under test. Using a BK Precision 880 LCR Meter at a test frequency of 100 kHz, 100 capacitance measurements were taken of both the air-filled capacitor and the dielectric-filled capacitor. All 100 capacitance measurements were averaged, and the dielectric constant was calculated. To verify the accuracy of the parallel plate capacitor test structure, the dielectric constant of 800 mm^3^ of DMSO was determined to be 45.6, which is less than a 3% discrepancy compared to the reported literature [9] and from the vendor’s datasheet. 

### 2.4. Transistor Characterization

Current and Voltage (I-V) measurements were conducted with an Agilent 4155C Semiconductor Parameter Analyzer and Semiprobe Probe Station under standard atmospheric and pressure conditions. Micromanipulator probes were suspended in the Galinstan droplets to act as the source and drain electrodes. The gate probe was then suspended in the dielectric. To avoid shorting the device, the micromanipulator probe was carefully lowered into the gate dielectric without touching the graphene surface.

## 3. Results and Discussion

**Figure 4:**
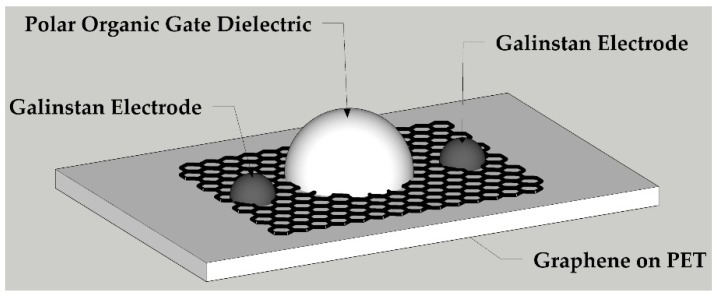
Visualization of the polar organic gated GFET.

To test the feasibility of liquid polar organic molecules as a gate material, a standard top-gated GFET architecture was adopted as illustrated in Figure 4.

Four different liquid polar organic molecules were used as organic gate dielectric materials for GFET devices. Their measured dipole moments and dielectric constants are compared in Table 1.

**Figure 5:**
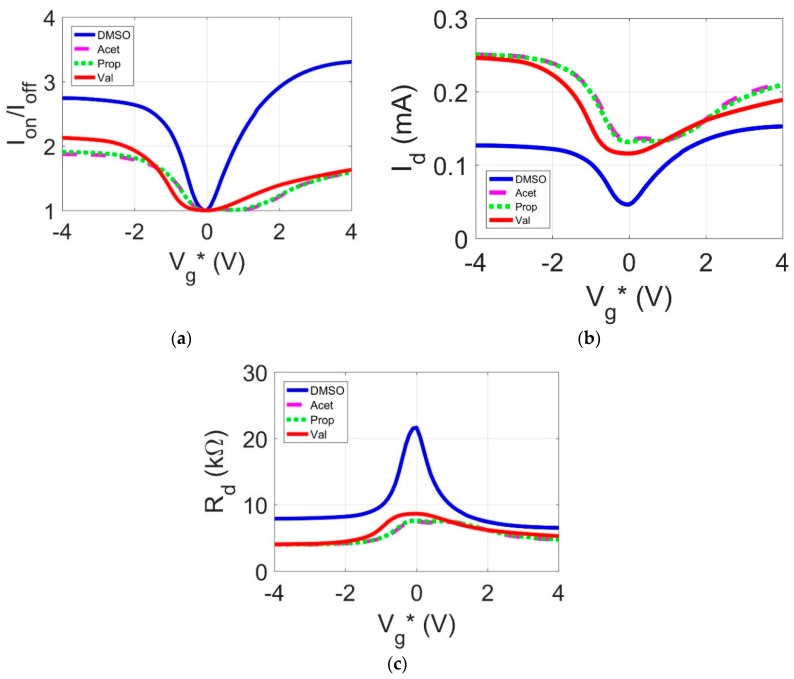
(**a**) I_on_/I_off_ as a function of normalized gate voltage (V_ds_ = 1 V) (**b**) Drain-to-source current (I_ds_) as a function of normalized gate voltage (V_d_ = 1 V); (**c**) Drain-to-source resistance (R_ds_)as a function of normalized gate voltage (V_d_ = 1 V).

**Figure 6:**
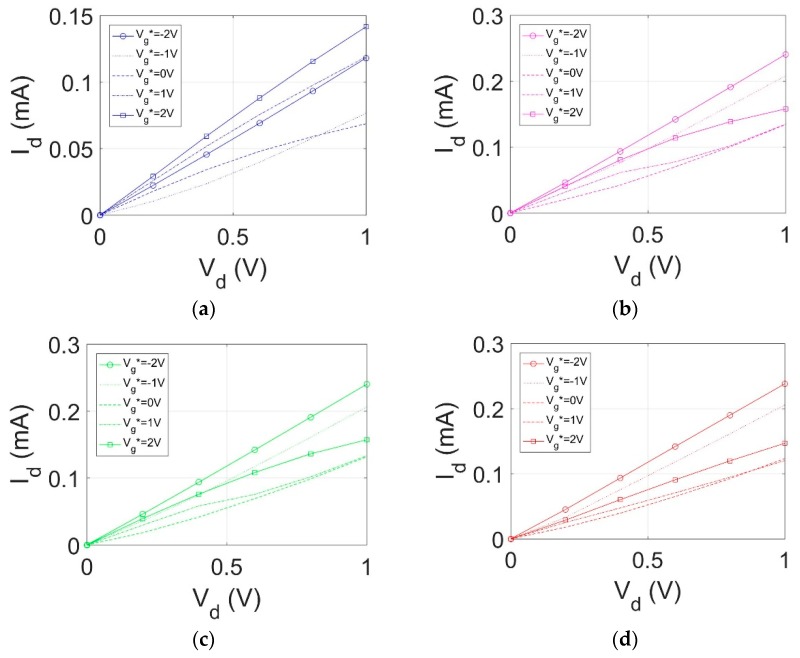
I_ds_ as a function of drain voltage for (**a**) DMSO, (**b**) Acetonitrile, (**c**) Propionamide, (**d**) Valeramide.

Ambipolar transport characteristics for the GFET devices with different liquid polar organic molecules as the gate material are shown in Figure 5 and Figure 6. Each transistor gate was varied from −4 V to 4 V and a drain-to-source voltage (V_ds_) was swept between 0.2 V to 1 V at 0.2 V intervals. The resulting GFET I-V curves for each polar organic dielectric are shown in relation to one another for comparison.

In Figure 5a–c, V_g_* = V_g_ − V_Dirac_ where Vg is the gate voltage and V_Dirac_ is the intrinsic Dirac point. Figure 5a shows the on-off current ratio for each of the transistors. This value demonstrates the ratio of the measured current at a given gate voltage over the minimum measured current. The values of V_g_* for the DMSO, acetonitrile, propionamide, and valeramide dielectrics are −0.4 V, 0.1 V, 0.1 V, and −0.6 V respectively. These values signify a shift in the charge carriers’ density brought on by the conduction of electrons through the dielectric. At V_g_*, the polar organic gated transistors experience zero charge carrier density. The negative Dirac point shift corresponds with a *p*-type doping profile while the positive shift in the Dirac point signifies an *n*-type doping profile. These values match the values for the electron and hole concentrations shown in Table 2. Both DMSO and valeramide have more hole charge carriers while propionamide and acetonitrile have more electron charge carriers.

From Figure 5b, the I_ds_-V_g_ relationship for the four dielectrics is shown with V_d_ = 1 V. The current magnitude of I_ds_ between −4 V < V_g_* < 4 V is larger than the current magnitudes as demonstrated in other flexible graphene-based transistors [5,16] at similar top gate voltage sweeps. Although the drain current magnitude presented in [17] is on the same order of magnitude, a gate voltage sweep between −40 V and 40 V was needed to produce this current magnitude. The higher drain current values corresponding to the polar organic dielectrics are related to the relatively low gate leakage current of all four organic gate dielectrics found in Table 3. The higher current magnitude at lower voltages means less power loss when devices are in their operating modes. This characteristic has many applications in a wide range of low-powered sensors.

The transfer characteristics shown in Figure 5b are asymmetric. This effect is caused by charge injection from the surface states of the dielectric into the graphene substrate. The resulting net charge on the graphene substrate causes *n*-type or *p*-type behavior. In addition, both [18,19] discuss at length the metal/graphene interface forming *p*-*n* junctions and pinning the Fermi level at the interface. This causes a different transfer characteristic for both the *n*-type and *p*-type charge carriers. Additionally, for the case of the amide-based solutions, an uneven dissolution of the polar organic molecule into the solvent may also be a cause of the asymmetry. When the polar organic molecule is not fully and uniformly dissolved, areas of charge concentrations appear. These pockets of charges lead to uneven charge injection into the graphene substrate, thus causing asymmetric transfer characteristics.

The data presented in Figure 5a shows that DMSO, the polar organic molecule with the highest dipole moment (4.06 D), has the largest average on-off current ratio while acetonitrile, the molecule with the lowest dipole moment (3.4 D), has the lowest average on-off ratio. This result is due to the higher polarity in DMSO, which creates a stronger field-effect in the graphene transistor. It is believed that propionamide and valeramide performed similarly despite the difference in dipole moments due to the increased water content used in the propionamide solution. Water possesses a considerably lower dipole moment than these polar organic molecules (1.85 D) and potentially offsets the stronger dipole moment of propionamide. Overall, the pure concentrations of DMSO and acetonitrile were confident markers showing that the on-off current ratios positively correlate with each molecule’s dipole moment, while the other two water-based solutions show there is flexibility to tune the field-effect through the dielectric’s weight/volume concentration.

Due to the observed relationships between the dipole moments and device transfer curves, it can be assumed that dipolar polarization—directly related to the dipole moment—is the dominant polarization effect in the given dielectric. Typically, dielectric constants, which signify the overall ability of a dielectric to distribute charge across it, are related to many different types of polarization including electron, ion, and dipolar polarizations [20]. However, in this case, it is clear that the strong dipole moments take precedence in these polar organic molecules.

To obtain various electrical properties of the GFETs, a graphene parameter extraction method [21] was used to calculate values for the electron and hole mobilities and charge carrier concentrations. These calculated values are shown in Table 2.

The characteristics observed in the presented data compare similarly to flexible GFETs with more typical dielectric materials and fabrication methods [5,16,17,22]. In addition, these devices were rapidly and inexpensively fabricated. Most of the tested polar organic molecules show an improved performance compared to other transistors in terms of current magnitude, charge carrier mobilities, and gate leakage current. For example, DMSO, acetonitrile, and the propionamide-water mixture show electron and hole mobilities on the same order as those found in [5,17,22]. In addition, the average drain current magnitude is also on the same order of magnitude as the previously cited papers. To further reduce the effects of gate leakage through the dielectric, the GFETs may be encased in a low gas permeable polymer such as polydimethylsiloxane (PDMS) or poly(methyl methacrylate) (PMMA). This would help reduce gate leakage because many of the polar organic dielectric materials are hygroscopic, meaning they absorb moisture from the atmosphere. The introduction of moisture into the dielectric causes increased electron conduction through the dielectric. Devices encased in PDMS or PMMA and fabricated in an atmosphere-controlled glove box will limit contamination and excess moisture, reducing gate leakage.

As with all electronic devices fabricated with liquid dielectrics, these polar organic gated GFET devices face high gate leakage currents. The average gate leakage currents for V_g_ < 0 V and 0 V < V_g_ are shown in Table 3. These values represent the arithmetic mean of all gate current values from gate voltages less than 0 V and greater than 0 V. The averages stated in Table 3 are lower than the gate leakage values reported in [5] by a whole order of magnitude. In addition, the data set shows that DMSO, the molecule with the highest dipole moment, also has the lowest average gate leakage current. In general, the polar organic dielectrics show less leakage than the commonly used ionic gel dielectrics reported in [5].

The results from this study show the feasibility of previously unexplored polar organic dielectrics for use in flexible GFETs. While polar organic dielectric GFETs lack the performance characteristics of oxide-based GFETs, they introduce a significantly more robust, flexible device with a quick, inexpensive fabrication process [6]. Furthermore, when coupled with graphene’s inherent sensing characteristics, organic molecule-gated graphene sensors have potential applications in chemical, biological, and optical sensing technologies. Graphene’s exceptional properties have already yielded fruitful studies in conformable humidity/moisture sensors, wideband photodetectors [23], applications in biomedical imaging, small molecule sensors, [24,25], and biological and chemical sensors [26,27]. This finding adds a flexibility feature that may be invaluable in the aforementioned applications which require dynamic sensors subjected to constant strain and flexure. 

## 4. Conclusions

For the first time, graphene-based transistors were fabricated using polar organic compounds as liquid gate dielectrics. We demonstrated that both the high dielectric permittivity and polarity of numerous polar organic compounds are ideal in creating an electric double layer-based field-effect transistor. Paired with the inherent ability of liquids to self-repair and to wet surfaces, polar organic dielectrics have the potential to become the dielectric at the heart of every flexible electronic device. Liquid dielectrics can be precisely printed without the need for expensive chemical vapor deposition and atomic layer deposition equipment. Paired with microfluidic channels, seemingly complex electronics can be easily implemented on a flexible substrate. Flexible electronics utilizing the outstanding mechanical properties of graphene could result in liquid gated GFETs leading to a new generation of high-performance sensors. To further the advancement of high performance flexible sensors, the authors encourage readers to explore several other polar organic molecules that are not explicitly mentioned in this article. A researcher can tailor polar organic molecules to chemically functionalize graphene to a particular chemical agent, should they so desire. In addition, researchers should utilize biomedical and microfluidic techniques to flow polar organic molecules in a sophisticated network of channels tailored to a specific sensor application. The intention of this study is to describe the new-found polar organic liquid alternatives to traditional rigid dielectrics, which can enable a variety of flexible sensor applications. 

## Figures and Tables

**Table 1:** Physical properties of organic dielectrics gated GFETs.

Organic Molecule	Dipole Moment (D) ^1^	Dielectric Constant
DMSO	4.06	46.7
Acetonitrile	3.40	37.5
Propionamide	3.85	117 ^2^
Valeramide	3.70	40 ^2^

^1^ Values for dipole moments were found in [12,13,14,15], and the National Institute of Standards and Technology (NIST)’s Computational Chemistry Comparison and Benchmark Database (CCCDB). Note that the dipole moment values for propionamide and valeramide represent a pure concentration of the organic molecule. ^2^ Due to the solid form of these organic molecules, they were mixed with distilled water at the listed concentrations. The experimental setup is explained in “Materials and Methods” section of the paper. Note: DMSO is dimethyl sulfoxide.

**Table 2:** Charge transport characteristics of graphene field effect transistors with different polar organic gate dielectrics.

Organic Molecule	Electron Mobility (cm^2^/Vs)	Hole Mobility (cm^2^/Vs)	Electron Concentration (10^−12^ cm^−2^)	Hole Concentration (10^−12^ cm^−2^)
DMSO	154	154.6	8.9	9.3
Acetonitrile	119.7	163.6	30.7	26.6
Propionamide-Water	54.7	68.8	8.9	6.8
Valeramide-Water	133.8	96.1	28.5	54

**Table 3:** Average gate leakage currents (V_g_) for V_g_ < 0 V and 0 V < V_g_.

Organic Molecule	Average Gate Current for V_g_ < 0 V (μA)	Average Gate Current for V_g_ > 0 V (μA)
DMSO	−2.91	1.59
Acetonitrile	−5.51	2.43
Propionamide	−5	2.19
Valeramide	−7.6	8.31
